# Interleukin-15 Treatment Induces Weight Loss Independent of Lymphocytes

**DOI:** 10.1371/journal.pone.0039553

**Published:** 2012-06-29

**Authors:** Nicole G. Barra, Marianne V. Chew, Sarah Reid, Ali A. Ashkar

**Affiliations:** Department of Pathology and Molecular Medicine, McMaster Immunology Research Centre and Institute for Infectious Disease Research, McMaster University, Hamilton, Ontario, Canada; Pennington Biomedical Research Center, United States of America

## Abstract

Obesity is a chronic inflammatory condition characterized by activation and infiltration of proinflammatory immune cells and a dysregulated production of proinflammatory cytokines. While known as a key regulator of immune natural killer (NK) cell function and development, we have recently demonstrated that reduced expression of the cytokine Interleukin-15 (IL-15) is closely linked with increased body weight and adiposity in mice and humans. Previously, we and others have shown that obese individuals have lower circulating levels of IL-15 and NK cells. Lean IL-15 overexpressing (IL-15 tg) mice had an accumulation in adipose NK cells compared to wildtype and NK cell deficient obese IL-15^−/−^ mice. Since IL-15 induces weight loss in IL-15^−/−^ and diet induced obese mice and has effects on various lymphocytes, the aim of this paper was to determine if lymphocytes, particularly NK cells, play a role in IL-15 mediated weight loss. Acute IL-15 treatment resulted in an increased accumulation of NK, NKT, and CD3^+^ T cells in adipose tissue of B6 mice. Mice depleted of NK and NKT cells had similar weight loss comparable to controls treated with IL-15. Finally, IL-15 treatment induces significant weight loss in lymphocyte deficient RAG2^−/−^γc^−/−^ mice independent of food intake. Fat pad cross-sections show decreased pad size with cytokine treatment is due to adipocyte shrinkage. These results clearly suggest that IL-15 mediates weight loss independent of lymphocytes.

## Introduction

Obesity is defined as an accumulation of adipose tissue and is associated with systemic chronic inflammation [1]. Altered immune responses in obese individuals have been recently linked to the development of comorbidities such as insulin resistance and dyslipidemia [1]–[3]. As an inflammatory condition, obesity is characterized by increased infiltration of various immune cells into adipose tissue, such as M1 polarized macrophages, as well as a decrease in anti-inflammatory immune cells such as M2 polarized macrophages [2], [4]. As prevalence rates and its associated chronic conditions continue to rise, insights into the pathophysiology related to obesity and the immune contribution to metabolic disease development is essential in formulating novel therapeutic strategies in treating obesity and its associated comorbidities.

Although several reports have examined the role of altered adipose macrophage phenotypes in obese individuals [2], [4], [5], recent studies have also demonstrated associations between obesity and alterations in lymphocyte populations. In obese adipose tissue, increased CD8^+^ T cells and decreased CD4^+^ and anti-inflammatory regulatory T cells have been found [6], [7]. Obese adipose tissue has been shown to activate CD8^+^ T cells leading to macrophage recruitment and activation [7]. As well, natural killer T (NKT) cells have been shown to play a role in metabolic abnormalities associated with obesity [8], [9]. Lastly, innate immune natural killer (NK) cells have reduced cytotoxicity in obese animals compared to lean controls [10]. Alterations in these lymphocyte populations in obese individuals suggest the presence or absence of these cell types may play a role in weight regulation. Since a dysregulation in the production of proinflammatory cytokines also contributes to this inflammatory state through such factors as interleukin-6 (IL-6) and tumor necrosis factor –α (TNF-α) [11], determining the role of these cytokines in regulating immune lymphocyte responses in obesity is also essential in formulating treatments for obese individuals.

While best known as a key regulator of immune natural killer cell function and development [12], [13], we and others have recently demonstrated that reduced expression of the cytokine Interleukin-15 (IL-15) is closely linked with increased body weight and adiposity in both mice and humans [14]–[19]. Previous work has shown that IL-15 treatment causes a significant decrease in adipose tissue mass in normal weight [19], [20] and obese rodents [18], reduces lipoprotein lipase expression [18], and may play a role in lipid oxidation [21], [22]. We have previously observed that over expression of IL-15 (IL-15 tg) in mice was associated with a lean body condition, while mice lacking IL-15 (IL-15^−/−^) gained significantly more weight, developing an obese phenotype when compared to control C57BL/6 (B6) mice. Also, we found that IL-15 tg mice had increased percentage of NK cells found in adipose tissue compared to normal weight B6 mice and their heavier NK cell deficient IL-15^−/−^ counterparts [14]. As well, obese individuals and mice placed on high fat diets have decreased NK cell numbers in circulation and in adipose tissue [14], [23]. We have also demonstrated that IL-15 treatment induces weight loss in diet-induced obese mice and in IL-15^−/−^ mice, which has also been shown to reconstitute NK cell populations [14], [24]. As well, IL-15 is known to activate other lymphocytes such as NKT cells [25], [26] and T cells [26], [27]. Whether NK cells and/or other lymphocytes contribute to the weight loss effects exerted by IL-15 treatment has yet to be determined.

In this study, we sought to determine if NK cells mediate weight loss in IL-15 treated animals. We first determined if NK cells, along with other lymphocytes such as NKT and T cells, accumulate in adipose tissue with acute IL-15 treatment in control B6 mice. In order to determine if IL-15 induces weight loss indirectly via NK cell activation, we depleted mice of NK cells using a NK1.1 cell depleting antibody, treated with IL-15, and monitored for weight loss. In order to determine the importance of lymphocyte activation via IL-15 treatment, we also employed the use of the RAG2^−/−^γc^−/−^ mouse model, which has no lymphocytes and lacks the γ-receptor subunit. Altogether, these experiments will determine the role of lymphocytes, specifically NK cells, in IL-15 regulation of adipose tissue.

## Methods

### Ethics

All animal experiments were approved by the Animal Research Ethic Board (AREB) of McMaster University. The AREB approval number is: 10-02-12.

### Animals

Sixteen week old C57BL/6 (B6) female mice were purchased from Charles River Laboratory (Quebec, Canada). Female lymphocyte deficient Balb/c RAG-2^−/−^γc^−/−^ mice were bred from breeding pairs given as a gift by M. Ito (Central Institute for Experimental Animals, Kawasaki, Japan) and maintained at McMaster University’s Central Animal Facility. Null mutation of the RAG2 gene prevents B and T lymphocyte development in these mice, while absence of the γ-chain subunit prevents NK cell maturation. Mice were caged in groups of five and maintained under controlled lighting (12∶12 L:D) and temperature (22°C) with *ad libitum* access to a low fat irradiated chow diet containing 18.6% protein, 6.2% fat, and 3.5% fiber (2918, Tekland Global Diets, Indianapolis, IN) and water.

### Delivery and Detection of IL-15 and NK1.1^+^ Cell Depletion Experiment

Body weights and food consumption were monitored daily from the onset of treatment until mice were sacrificed. Food consumption was measured for a period of five consecutive days. Preweighed food was placed in food hoppers and measured daily on a per-cage basis. Food intake was recorded as grams consumed per gram of mouse per day. Percentage of weight loss was recorded when mice were delivered either an Ad-expressing human IL-15 vector, Opt.hIL-15 (“AdIL-15”), or an empty adenoviral vector (“AdControl”) as previously described [28]. Briefly, an 18-aa optimized signal peptide was inserted upstream of a mature hIL-15 gene by multi-step polymerase chain reactions (PCRs) using primers provided by the Molecular Biology Institute (McMaster University). After the 402-bp PCR product was isolated (Opt.hIL-15), cloned into expression vectors using *KpmI* and *XhoI* sites, Opt-hIL-15 was inserted into the adenoviral shuttle vector pDC316 and subsequently generated Ad-Op-hIL-15 pDC316 in the Robert E. Fitzhenry Vector Laboratory (McMaster Immunology Research Centre, McMaster University) [28].

B6 mice and RAG-2^−/−^γc^−/−^ mice were administered with 5×10^8^ pfu AdIL-15, AdControl, or 200 µl PBS via IV tail injections on day 0, 2, and 4 (n = 5 per group). For the NK1.1^+^ cell depletion experiment, B6 mice were injected intraperitoneally with 200 µg of anti-mouse NK1.1 antibody (PK136 mouse immunoglobulin G2a hybridoma HB191; ATCC) daily for two days prior to AdIL-15 treatment and subsequently every three days post treatment (n = 7 per group). Mice were anaesthetized and sacrificed on day 8. Prior to sacrifice, blood was collected through the abdominal aorta and centrifuged at 5,000 rpm for 10 minutes to collect serum. Human IL-15 levels were quantified from serum using hIL-15 DuoSet ELISA kit (R&D Systems, Minneapolis, MN, USA).

### Adipose Tissue Collection, Histology, and Cell Quantification

Visceral gonadal fat pads from RAG-2^−/−^γc^−/−^ mice were weighed and fixed in paraformaldehyde. Tissues were then embedded in paraffin, and two cross-sections per mouse were stained with hematoxylin and eosin (H&E). For each cross section, 8 fields of view were quantified for cell areas using AxioVision software (created by Carl Zeiss MicroImaging).

### Cell Isolation from Fat Tissue for FACS Analysis

Weighed visceral gonadal fat pads from B6 mice were isolated, washed in PBS, minced, and digested in collagenase A (Roche Applied Science, Laval, Quebec, Canada) and 0.025 mg/ml DNase (Roche Applied Science, Laval, Quebec, Canada) for 30 min at 37°C. The cell suspension was filtered through a 100 µm, followed by a 70 µm and 40 µm cell strainer to remove tissue particulate. This solution was then centrifuged for 10 min at 1,200 rpm at 4°C. The supernatant, which included any remaining adipocytes, was discarded and the stromal vascular fraction was resuspended in ACK lysis buffer to remove red blood cells, and washed in PBS. Cells were counted using a hemocytometer and resuspended in a 0.2% bovine serum albumin PBS solution for FACS analysis. Five-hundred thousand cells per well were plated in a 96 well plate. After cells were washed and blocked using CD16/CD32 antibody (eBioscience, San Diego, CA), they were surfaced stained with fluorescein isothiocyanate–, alexa fluor 700-, and phycoerythrin-conjugated anti-mouse CD45.2 (clone 104, eBioscience), CD3 (clone 17A2, eBioscience), and NK1.1 (clone PK136, BD Pharmingen) antibodies, respectively. Stained cells were analyzed on a LSRII flow cytometer collecting 50,000 gated events and FlowJo flow cytometry analysis software.

### Statistics

All statistical analyses were performed using Graph Pad Prism 4. The results are expressed as mean ± SEM. Data were analyzed using one-way ANOVA followed by Tukey’s *post hoc* multiple comparisons test for 3 group comparisons, while Student *t*-test was used for 2 group comparisons. Significance is indicated when p<0.05.

**Table 1:** Characteristics of mice before and after IL-15 treatment.

	Treatment Groups		
Measure	PBS	AdControl	AdIL-15
Before Treatment(B6 Mice)	25.80±1.55	26.34±0.78	25.32±0.82
After Treatment(B6 Mice)	25.82±1.55	26.40±0.78	22.35±0.84***
Before Treatment(Rag2^−/−^γ_c_^−/−^ Mice)	40.62±2.76	37.72±1.47	39.36±3.61
After Treatment(Rag2^−/−^γ_c_^−/−^ Mice)	41.31±2.71	37.77±1.58	30.91±2.76**

Data are presented as mean ± SEM. Body weights expressed as grams. Asterisks used to denote significance using a paired Student t-test comparing before and after body weights within the same group.

** P<0.01,

*** P<0.001.

**Figure 1:**
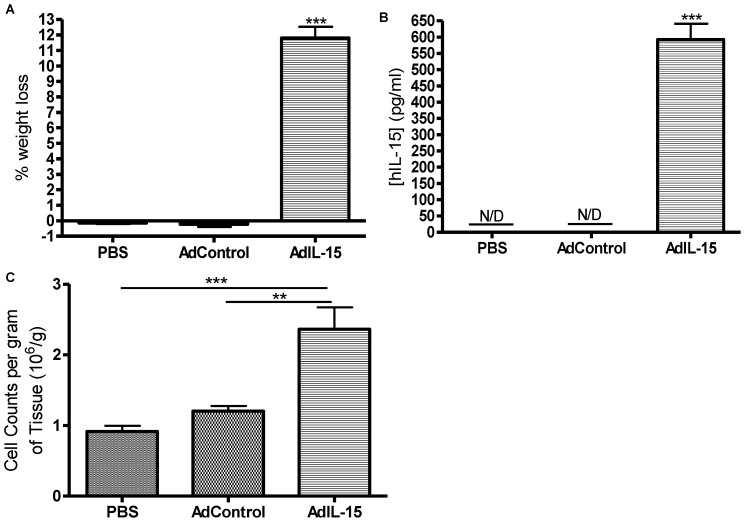
Acute interleukin-15 administration results in weight loss and increased cell number in visceral adipose tissue of B6 mice. (**A**) Bar graph shows percentage of weight loss in female B6 mice treated i.v. with either phosphate-buffered saline (PBS), an empty adenoviral construct not expressing IL-15 (Ad-Control), or IL-15 expressing adenoviral construct (AdIL-15). (**B**) Serum collected on day 8 was analyzed for IL-15 expression using a hIL-15 DuoSet ELISA kit. (**C**) The cells referred to are the number of cells in the stromal vascular fraction from digested adipose tissue in each group. The y-axis refers to the number of cells (×10^6^) counted from the stromal vascular fraction per gram of digested visceral gonadal adipose tissue shown in bar graph (n = 5 per group). **P<0.01, ***P<0.001.

## Results

### Acute IL-15 Treatment Results in an Accumulation of NK, NKT, and T Cells in Adipose Tissue

**Figure 2:**
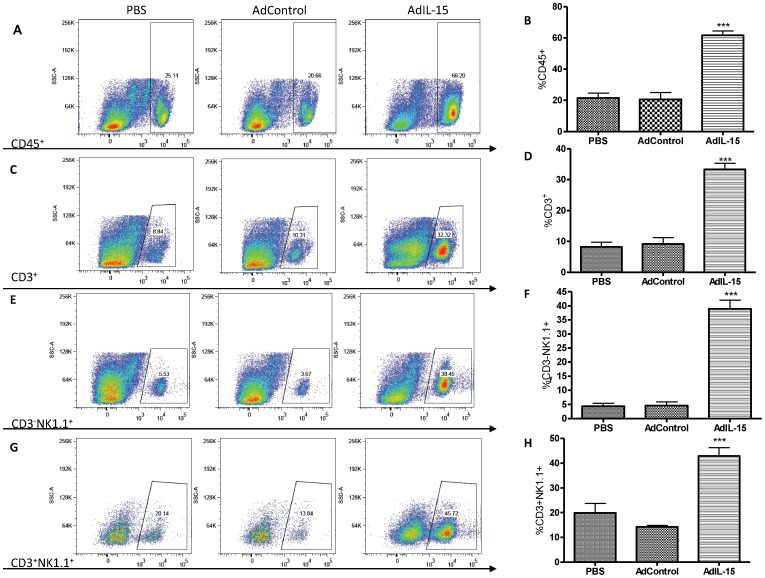
Acute interleukin-15 treatment results in CD45^+^, NK, NKT, and CD3^+^ T cell accumulation in visceral adipose tissue in B6 mice. Analysis of (**A**) CD45^+^, (**C**) CD3^+^ (T), (**E**) CD3^−^NK1.1^+^ (NK), and (**G**) CD3^+^NK1.1^+^ (NKT) cell populations in visceral gonadal adipose tissue from PBS, AdControl, and AdIL-15 treated animals. The percentage of (**B**) CD45^+^, (**D**) CD3^+^ (T), (**F**) CD3^−^NK1.1^+^ (NK), and (**H**) CD3^+^NK1.1^+^ (NKT) cell populations are represented as a bar graphs (n = 5 per group). ***P<0.001.

Since IL-15 is inextricably linked to NK cell function, we wanted to determine if an association between IL-15 mediated weight loss and an accumulation of NK cells in visceral gonadal fat occur in B6 mice given an acute dose of IL-15. Sixteen week old B6 mice had similar body weights between groups prior to treatments (Table 1). Mice treated with AdIL-15 lost significantly more weight, had sustained serum hIL-15 expression, and had higher cell numbers in the stromal vascular fraction of digested visceral fat on a per gram basis compared to mice given either AdControl or PBS (Figure 1A–C). Flow cytometric analysis revealed that IL-15 treated animals had an increase in the percentage of leukocytes (CD45^+^), T (CD3^+^), NK (CD3^−^NK1.1^+^) and NKT (CD3^+^NK1.1^+^) cells in visceral adipose tissue compared to controls (Figure 2). Similarly, peripheral leukocytes, T, NK, and NKT cells were also significantly elevated in spleens of IL-15 treated mice compared to controls (data not shown).

### IL-15 Treatment Induces Weight Loss Independent of NK and NKT Cells

**Figure 3:**
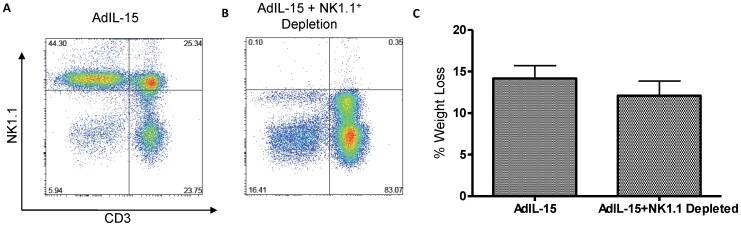
Treatment with interleukin-15 induces weight loss in NK1.1^+^ cell depleted B6 mice. Analysis of CD3 and NK1.1 cells gated from a CD45.2^+^ population in (**A**) AdIL-15 treated B6 mice and (**B**) NK1.1^+^ depleted AdIL-15 treated mice in visceral gonadal adipose tissue. (**C**) Bar graph shows weight loss in female treated mice (n = 7 per group).

Since IL-15 treatment results in an accumulation of NK cells in adipose tissue in B6 mice (Figure 2), we wanted to verify whether weight loss was a direct effect of IL-15 or mediated indirectly by activated NK cells. In figure 3, we show that the NK1.1^+^ cell depleting antibody is effective in depleting NK and NKT cells from adipose tissue (Figure 3A, B). We found no significant difference in the percentage of weight loss in B6 mice treated with AdIL-15 compared to those depleted of NK and NKT cells (Figure 3C, Table 2).

**Table 2:** Characteristics of mice before and after IL-15 treatment.

	Treatment Groups	
Measure	AdIL-15	AdIL-15+NK1.1 Depletion
Before Treatment(B6 Mice)	29.18±2.65	29.34±1.84
After Treatment(B6 Mice)	25.09±2.49**	25.91±1.93***

Data are presented as mean ± SEM. Body weights expressed as grams. Asterisks used to denote significance using a paired Student t-test comparing before and after body weights within the same group.

** P<0.01,

*** P<0.001.

### Interleukin-15 Treatment Induces Weight Loss in the Absence of Lymphocytes

**Figure 4:**
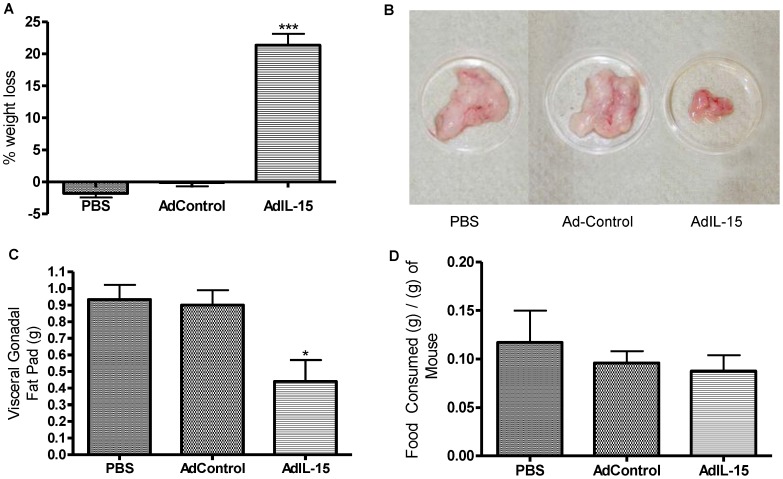
IL-15 induces weight loss independent of lymphocytes and in the absence of the common gamma chain (γc). (**A**) Percentage of weight loss represented as a bar graph. (**B**) Picture shows excised visceral gonadal fat taken from representative female naïve, Ad-Control, and AdIL-15 treated RAG2^−/−^γc^−/−^ mice. (**C**) Visceral gonadal fat pad in grams shown as a bar graph (n = 5 per group). (**D**) Food consumed per gram of mouse monitored for 5 consecutive days (n = 5 per group). *P<0.05, ***P<0.001.

Since acute IL-15 treatment resulted in an accumulation of other lymphocytic cells such as T cells, we utilized lymphocyte deficient RAG2^−/−^γ_c_^−/−^ mice. These mice had similar body weights between groups prior to treatment (Table 1). Mice treated with AdIL-15 lost significantly more weight compared to AdControl or PBS treated animals (Figure 4A). Treatment of these mice with AdIL-15 clearly showed a marked difference in the size and weight of the abdominal visceral gonadal fat pad compared to naïve and vehicle controls (Figure 4B, C). These differences were not attributed to altered food consumption (Figure 4D).

### Acute IL-15 Administration Leads to Less Fat Deposition

**Figure 5:**
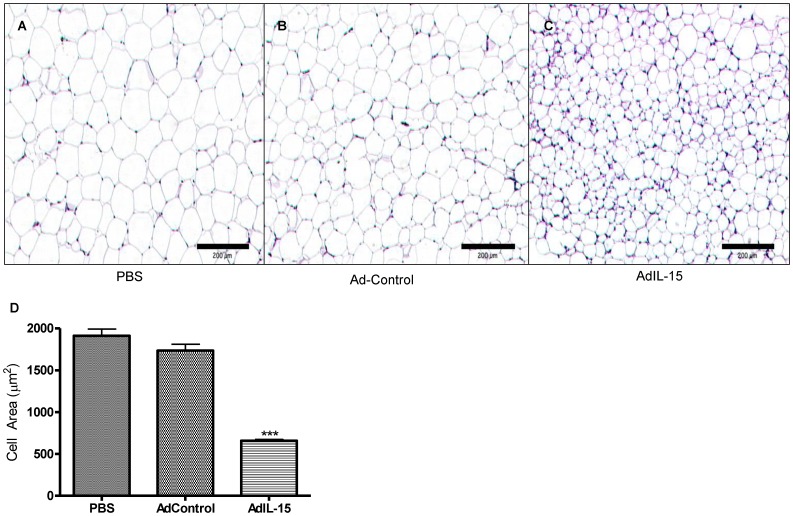
IL-15 treatment affects adipocyte size in RAG2^−/−^γc^−/−^ mice. Representative cross sections of visceral gonadal fat pads excised from RAG2^−/−^γc^−/−^ mice treated i.v. with (**A**) PBS, (**B**) AdControl, or (**C**) AdIL-15 were stained with hematoxylin and eosin (H&E). (**D**) Bar graph shows cell areas determined under 5× objective from each group determined using AxioVision software (n = 5 per group). ***P<0.001.

Since we observed that IL-15 has significant effects on fat mass (Figure 4), we then examined cross sections of abdominal fat pads to assess if IL-15 treatment may be attributed to decreased cell size and fat content. These sections reveal shrunken adipocytes resulting in a lower cell area in AdIL-15-treated RAG2^−/−^γ_c_^−/−^ mice compared to both the Ad-Control and PBS groups (Figure 5). This suggests that the effects of IL-15 on adiposity are not mediated by lymphocytes and occur independently of signaling through the common-γ-chain.

## Discussion

Results from this paper show that acute IL-15 administration results in weight loss and an accumulation of NK, NKT, and CD3^+^ T cells in visceral adipose tissue in B6 mice. Previously, we have shown that IL-15 tg mice, which remain lean over time, have a significant increase in the percentage of NK cells found in adipose tissue compared to control B6 mice and IL-15^−/−^ mice [14]. Since an accumulation of NK cells is associated with lean body weight and the effects of IL-15 are inextricably linked with NK cell function, we utilized a NK1.1 depleting antibody to determine whether IL-15 mediates weight loss directly or indirectly through NK and/or NKT cells in B6 mice. To determine if IL-15 mediates weight loss through other lymphocyte populations such as T cells, we utilized the lymphocyte deficient RAG2^−/−^γc^−/−^ mouse model. IL-15 treatment resulted in weight loss, decreased visceral fat weight, and shrunken adipocytes in RAG2^−/−^γc^−/−^ mice suggesting that this cytokine may directly affect adipocytes by signaling through other receptor subunits excluding the common-γ-chain.

Immune cell populations and their contribution to metabolic disease development in obesity have garnered a great deal of interest; however, whether immune cells play a role in weight regulation and adiposity has not been thoroughly examined. Lymphoid cells are in close proximity to adipocytes in various locations such as bone marrow, perinodal adipose tissue surrounding lymph nodes, and subcutaneous and visceral adipose sources [29], [30]. This close contact provides opportunity for cross talk between these two cell types. Secretion of various adipokines from adipose demonstrate that this tissue may directly influence lymphocyte activity [6], [7], [31]–[33]. We were interested in determining whether factors that regulate fat mass, such as IL-15, influence lymphocytes within adipose tissue and affect adipocyte size and fat mass through their activation. The effects of IL-15 on NK, NKT, and T cells include: to promote the development, survival, and activation of NK and NKT cells [13], [25], [26], [34], [35]; control the induction of CD4^+^ memory T cells, as well as the survival and proliferation of activated naïve and memory CD8^+^ T cells [26], [27], [34]; and induce interferon-gamma (IFN-γ) production, cytotoxicity, and perforin/granzyme expression in both NK cells and CD8^+^ T cells and TNF-α in CD8^+^ T cells [12], [25], [35], [36]. Nevertheless, the actual function of these cell types in lean adipose tissue is unknown.

*In* vitro studies have shown that IL-15 directly affects adipose tissue using cultured adipocytes. Recombinant IL-15 treatment was shown to inhibit preadipocyte differentiation and lipid deposition using the murine adipogenic 3T3-L1 cell line [37]. Similar results were shown in lipoaspirate-derived human adipocytes treated with IL-15 at time of differentiation [14]. Inhibition of preadipocyte differentiation is associated with increased mRNA expression of calcineurin [38] and/or alterations in Signal transducers and activator of transcription 5 (STAT5) expression [39]. As well, IL-15 administration has been recently shown to affect lipid content in mature differentiated adipocytes *in vitro*, suggesting that IL-15 directly affects adipocytes independent of lymphocytes [39].

It has been previously shown, using ribonuclease protection assays, that mRNA for all three IL-15 receptor signaling subunits (α, β, γ) are present in white adipose tissue, suggesting that circulating IL-15 is capable of directly signaling in adipocytes [18]. IL-15 signals through a heterotrimeric receptor complex, including a β subunit shared with IL-2 and a γ-chain subunit shared with other interleukins (IL-2, -4, -7, -9, and -21). The third unique α subunit confers specificity and high affinity binding of IL-15 to this complex [40], [41]. Studies have shown that IL-15 signals differently in immune lymphocytes compared to myleiod populations [42,43, Reviewed in 41]. The mechanism of IL-15 signaling in adipocytes is currently unknown.

The lymphocyte deficient RAG2^−/−^γ_c_^−/−^ mouse model lacks mature NK cells due to the absence of the receptor γ-chain subunit, which is required for NK cell maturation. The importance of signaling through the receptor γ-chain has been previously examined in other animal models. Alvarez *et al.* showed IL-15 treatment reduced white adipose tissue mass without altering food intake in leptin-deficient ob/ob mice but not in leptin receptor-negative fa/fa Zucker rats. These authors determined that IL-15 did not effect adipose tissue in these obese rats due to a down regulation in receptor γ-chain expression [18]. Our data in RAG2^−/−^γ_c_^−/−^ mice, on the other hand, demonstrates that the receptor γ-chain is not required for IL-15 to induce weight loss and reduce adipocyte cell size. This suggests that the γ-chain receptor subunit is dispensable for IL-15 signaling in adipocytes or compensatory mechanisms may occur in its absence. Although the receptor γ-chain has been shown to be indispensible for NK and NKT cell development [32], [44], we have previously shown that IL-15 may mediate its effects in the absence of this receptor subunit resulting in anti-tumor and anti-viral activity [28], [45], as well as in activating myeloid immune cells [46].

Since IL-15 has been shown to decrease white adipose mass [19], we believe that acute IL-15 administration may result in direct lipolysis of adipose tissue, resulting in the release of free fatty acids into circulation. Alemendro *et al*. [22] previously showed that the administration of IL-15 in rats resulted in increased whole body and skeletal muscle fatty acid oxidation. Increased mRNA expression of several genes involved in fatty acid oxidation including carnitine palmitoyltransferase II (CPT-II) and peroxiome proliferator-activated receptor- δ (PPAR-δ) in the liver and skeletal muscle, respectively, demonstrates that IL-15 may induce the breakdown of fatty acids at peripheral tissues. Whether IL-15 treatment promotes altered mitochondrial function by increasing fatty acid usage in the liver and skeletal muscle needs to be determined. Another possible mechanism in which IL-15 may exert its weight loss effects is through the activation of myeloid derived cells. Interleukin-15 expression has been reported to alter macrophage activity in IL-15 tg and IL-15^−/−^ mouse models [28], [47]. Adipose tissue macrophages have been shown to alter insulin sensitivity in adipose tissue [1]. Recently, M2 polarized macrophages have been found to directly induce lipolysis through the release of catecholamines in white adipose tissue [48]. Whether or not IL-15 treatment influences macrophage polarization and possible secretion of lipolytic factors within white adipose tissue has yet to be examined. Lastly, rats given a single intravenous injection of this cytokine had a significant decrease in triglyceride absorption, without affecting gastric emptying and intestinal mobility [49]. Therefore, altered intestinal lipid absorption may in part explain the anti-obesity effects of IL-15.

In conclusion, our data suggest that IL-15 treatment results in an accumulation of NK, NKT, and CD3 T lymphocytes in adipose tissue. However, IL-15 mediates weight loss independent of lymphocyte activation and signaling through the common γ-chain. There is currently a lack of knowledge regarding the effects of IL-15 on metabolic tissues. Our findings have clear implications in the field of immuno-metabolism by demonstrating the importance of immune factors, like IL-15, in regulating adipose tissue mass. Future studies should continue to focus on determining the mechanism(s) in which IL-15 affects adipocytes.
